# Survival rate of ovarian cancer in Asian countries: a systematic review and meta-analysis

**DOI:** 10.1186/s12885-023-11041-8

**Published:** 2023-06-16

**Authors:** Zahra Maleki, Mohebat Vali, Hossein-Ali Nikbakht, Soheil Hassanipour, Aida Kouhi, Saman Sedighi, Roya Farokhi, Haleh Ghaem

**Affiliations:** 1 Student Research Committee, Shiraz University of Medical Sciences, Shiraz, Iran; 2 Social Determinants of Health Research Center, Health Research Institute, Department of Biostatistics & Epidemiology, School of Public Health, Babol University of Medical Sciences, Babol, Iran; 3 Gastrointestinal and Liver Diseases Research Center, Guilan University of Medical Sciences, Rasht, Iran; 4 Department of Pathology, Keck School of Medicine, University of Southern California, Los Angeles, CA USA; 5 Department of Neurosurgery, Keck school of Medicine, University of Southern California, Los Angeles, CA USA; 6 Department of Health, Health Systems Research, Health Research Institute, Babol University of Medical Sciences, Babol, Iran; 7 Non-Communicable Diseases Research Center, Shiraz University of Medical Sciences, Shiraz, Iran; 8 Department of Epidemiology, School of Health, Shiraz University of Medical Sciences, Shiraz, Iran

## Abstract

**Background:**

Ovarian cancer is amongst one of the most commonly occurring cancers affecting women, and the leading cause of gynecologic related cancer death. Its poor prognosis and high mortality rates can be attributed to the absence of specific signs and symptoms until advance stages, which frequently leads to late diagnosis. Survival rate of patients diagnosed with ovarian cancer can be used in order to better assess current standard of care; the aim of this study is to evaluate the survival rate of ovarian cancer patients in Asia.

**Methods:**

Systematic review was performed on articles that were published by the end of August 2021 in five international databases, including Medline / PubMed, ProQuest, Scopus, Web of Knowledge, and Google Scholar. The Newcastle-Ottawa quality evaluation form was used for cohort studies to evaluate the quality of the articles. The Cochran-Q and I^2^ tests were used to calculate the heterogeneity of the studies. The Meta-regression analysis was also done according to when the study was published.

**Results:**

A total of 667 articles were reviewed, from which 108 were included in this study because they passed the criteria. Based on a randomized model, the survival rates of ovarian cancer after 1, 3 and 5 years were respectively 73.65% (95% CI, 68.66–78.64), 61.31% (95% CI, 55.39–67.23) and 59.60% (95% CI, 56.06–63.13). Additionally, based on meta-regression analysis, there was no relationship between the year of study and survival rate.

**Conclusions:**

The 1-year survival rate was higher than that of 3- and 5-year for ovarian cancer. This study provides invaluable information that can not only help establish better standard of care for treatment of ovarian cancer, but also assist in development of superior health interventions for prevention and treatment of the disease.

**Supplementary Information:**

The online version contains supplementary material available at 10.1186/s12885-023-11041-8.

## Background

Cancer is amongst the most common causes of death globally and is predicted to be a major contributor to poor quality of life in the 21st century [1, 2]. Ovarian cancer is commonly occurring cancers among women. It has been estimated that 190,000 new cases of ovarian cancer are reported annually worldwide, and its incidence is more prevalent in developed countries. In most cases, because of lack of specific signs and symptoms and absence of proper screening, ovarian cancer is detected in later stages of the disease, which often leads to poor outcomes [3]. While the incidence rate of ovarian cancer is less than breast cancer, it is estimated to be three times more lethal, and by the year 2040, its mortality rate is predicted to increase significantly [4].

Ovarian cancer incidence and epidemiology patterns vary globally and are correlated with various risk factors that can contribute to development of the disease [5]. Non-Hispanic white women have been reported to have the highest prevalence of ovarian cancer (12.0 per 100,000), followed by Hispanic women (10.3 per 100,000), non-Hispanic blacks (9.4 per 100,000), and Asian / Pacific Island women (9.2 per 100,000). Additionally, ovarian cancer mortality has a different global pattern, and is the highest amongst black women, which is mostly due to the differences in prevention, diagnostic and treatment strategies [6].

There is growing evidence to suggest that the management of ovarian cancer should be personalized, taking into account the patient’s performance status. [7]. It is essential to be able to predict the incidence rate of ovarian cancer and its survival rate given this information can help develop and enhance strategies and interventions for prevention and early diagnosis of the disease. The survival rate of ovarian cancer is related to many factors, including the stage and degree of disease, age, histology, appropriate surgical treatment, appropriate chemotherapy, and tumor site [8]. Furthermore, several risk factors can contribute to the development of cancer. Identifying and addressing these risk factors can potentially aid in cancer prevention. Moreover, elucidating the mortality rates of the disease, and global incident patterns can help develop strategies aimed at prevention. Currently, there are no comprehensive studies on the survival rate of ovarian cancer in Asian countries. Therefore, this systematic review and meta-analysis were conducted to determine the survival rate of ovarian cancer in Asian countries.

## Mehtods

The present study is a systematic review and meta-analysis of ovarian cancer survival rate. The method by which the present study is reporting is based on the PRISMA (Preferred Reporting Items for Systematic Reviews and Meta-Analysis) checklist [9].

### Search Strategy

In this study, authors surveyed five databases: Medline / PubMed, ProQuest, Scopus and the Web of Knowledge and Google scholar for grey literature and included studies published by the end of August 2021. Keywords that were selected to search databases included.

Ovarian Neoplasms [Mesh], Survival OR Survival Analysis OR Survival Rate, Asian Countries (Names of countries) (Appendix 1).

The data that were collected were entered onto EndNote, X7 software and duplicated articles were deleted. Two researchers examined the articles independently, using search strategies that are presented in Appendix 1.

### Inclusion and exclusion criteria

This study included observational studies (cross-sectional, case-control, and cohort studies) on ovarian cancer survival that were published by the end of August 2021 and were published in English. Review and meta-analysis studies or studies that did not report sample size or survival confidence interval were excluded.

### Quality evaluation and data extraction

The Newcastle-Ottawa Quality Assessment Checklist was employed to consider the quality of the selected manuscripts [10].

Two investigators performed the initial search of the studies. After screening studies and extracting results, the quality of the manuscripts were determined independently by two other investigators. If the two investigators were in disagreement, the preselected leader of the team would give their final opinion on the article.

All articles, which were included in this study were selected from a pre-determined checklist. This checklist encompassed the author’s name, publication year, study period, sample size, cancer type, country, and survival rates of 1, 3, and 5 year. Data extraction was done independently by two researchers.

### Statistical analysis

The Cochran Q test (with a significance level of less than 0.1) and I^2^ statistics were used to determine the heterogeneity between studies. In the presence of heterogeneity, the Random-Effects Model was used by the Inverse-Variance Method, and if there was no heterogeneity, the fixed effects model was used. Meta-regression analysis and subgroup analysis were used in case of heterogeneity between studies. Analysis was performed on STATA software version 16 and MEDCALC version 14.

### Additional analysis

The year in which the study was published was utilized in Meta-Regression analysis due to the high heterogeneity of the studies.

### Bias Risk among Studies

The Random Effects Model is utilized to reduce the risk of bias in studies [11, 12]. Egger diffusion bias evaluation test was also utilized to determine the risk of diffusion bias (publication bias) [13].

## Results

### Study selection

**Fig. 1:**
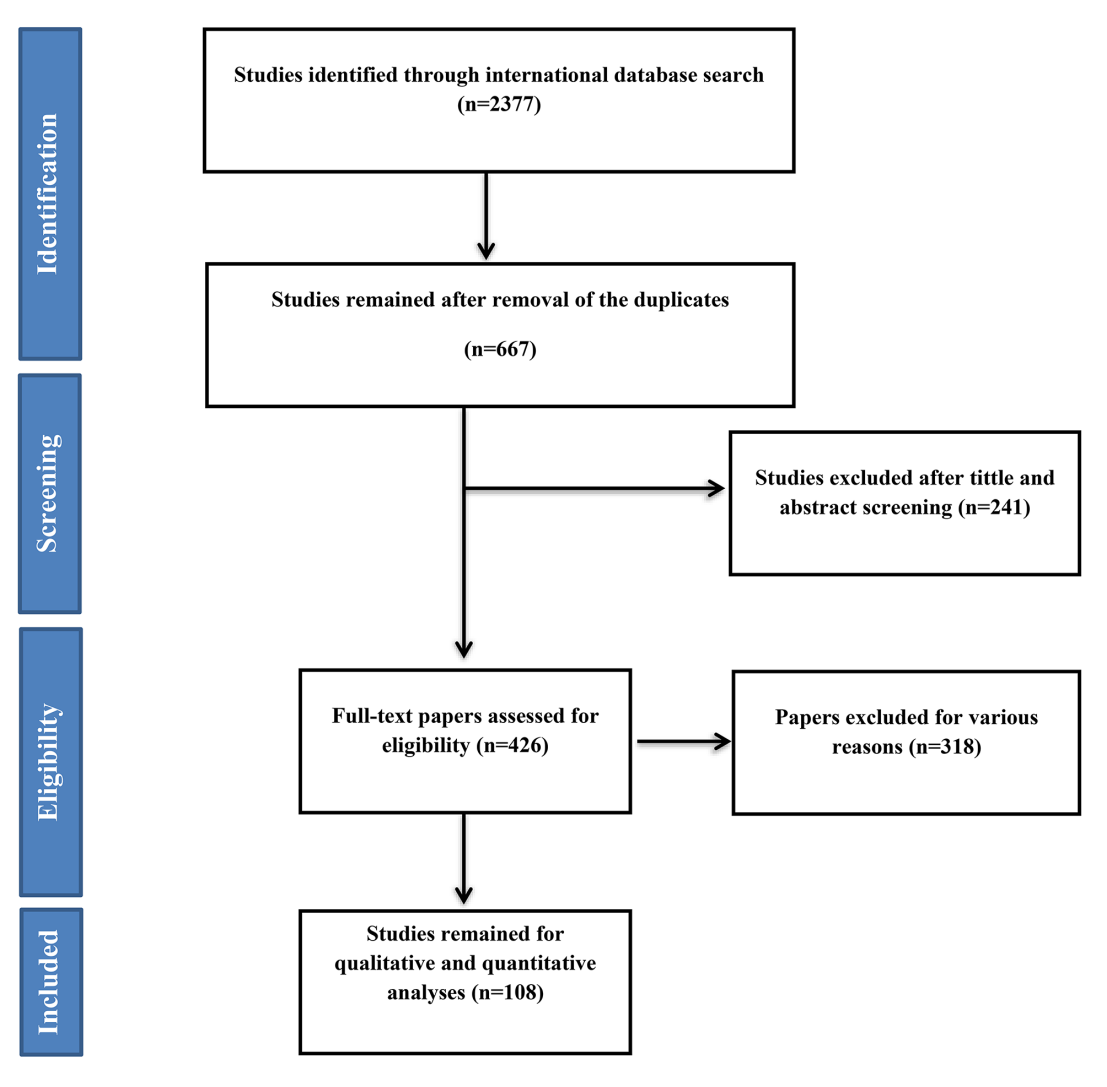
Flowchart of the included eligible studies in systematic review

2377 articles were found after searching all international databases, and after omitting duplicated articles, 667 articles were included in the review stage. After careful examination of the titles and abstracts of the selected articles, 426 articles were considered for the next step. At this stage, the full text of the articles was reviewed, and 108 retrospective cohort articles were part of the final analysis. The references of imported articles were also reviewed to add applicable studies. The study selection process is shown in Fig. 1.

### Study characteristics

The articles were chosen from January of 1989 to August 2021. 108 studies over the mentioned period were eligible. 56 articles from China, 13 articles from Hong Kong, 21 papers from India, 13 articles from Iran, 2 articles from Israel, 57 articles from Japan, 29 papers from South Korea, 2 papers from Kuwait, 16 papers from Singapore, 11 articles from Taiwan, 16 papers from Thailand, and 14 articles from Turkey were reviewed. Descriptive information for these studies is provided in Table 1. The median follow-up period in the studies was 9 years.

**Table 1:** Basic Information of Included Studies

Order	Author (year)	Location	Time period	Sample size	Ovary Survival Rate		
					1-year survival rate	3-year survival rate	5-year survival rate
1	Khalafi-Nezhad A,2021	Iran	2001–2016	132	93.80%	85.20%	66.00%
2	Aoki D.2014	Japan	2005–2011	4672	-	-	92.00%
3	Arab, M.2009	Iran	2000–2004	1246	-	-	61.00%
4	Ayhan A.2008	Turkey	1982–2004	91	-	-	83.00%
5	Bhika B.2004	India	1992–1994	4865	51.90%	29.00%	23.80%
6	Bi R.2016	China	1999–2009	73	-	-	78.80%
7	Bian,C.2015	China	2005–2010	339	-	-	25.00%
8	Bozkaya, Y.2017	Turkey	2000–2013	52	47.00%	-	-
9	C.Li.2012	China	2000–2005	131	-	-	39.14%
10	Chay W.Y.2013	Singapore	1963–2012	133	-	-	75.40%
11	Chen C.A.2018	Taiwan	2000–2011	510	-	-	84.60%
12	Chen S.2014	China	2008–2010	107	-	30.90%	41.80%
13	Chen, J. G. 2011	China	1992–2000	101	51.30%	34.70%	32.70%
14	Chen,M.C.2018	Taiwan	1979–2008	121	87.00%	-	61.00%
15	Chen,Y 0.2015	China	2001–2011	816	-	-	59.00%
16	Chia, K. S., 2011	Singapore	1968–1997	718	81.80%	67.50%	61.20%
17	Chia, K. S., 2001	Singapore	1968–1972	901	44.00%	44.00%	52.00%
18	Chul Chun,K.2011	Singapore	1989–2008	40	-	-	-
19	Chung,H.H.2007	South Korea	1993–2002	4778	89.10%	72.50%	65.90%
20	Dan Nie.2019	China	2008–2014	178	-	-	41.30%
21	Dikshit, R. 2011	India	1991–1995	69	63.80%	47.80%	18.80%
22	E. Alawadhi,2019	Kuwait	2005–2009	221	79.00%	-	42.60%
23	Egemen Ertas,I.2014	Turkey	1995–2010	42	-	-	81.40%
24	Gaemmaghami, F.2011	Iran	1998–2008	186	-	42.00%	43.0%
25	Gaurav Das,2020	India	2005–2012	958	92.50%	92.50%	92.50%
26	Gek-Hsiang Lim.2009	Singapore	1978–1982	1422	-	-	64.70%
27	Ghaemmaghami, F.2008	Iran	1997–2004	21	-	-	39.00%
28	Guangquan Liu.2017	China	2001–2017	32	-	-	30.00%
29	Gue,J.2018	China	2003–2016	143	-	-	96.80%
30	Guvenal, T.2013	Turkey	2001–2013	539	-	-	99.00%
31	Han,Y.2016	China	2000–2015	136	-	-	92.10%
32	Hee-Beom Yang,2020	South Korea	1995–2016	80	-	-	57.90%
33	Helpman, L.2005	Israel	1995–2011	71	-	-	68.00%
34	Higash, M.2011	Japan	1986–2008	1582	-	-	91.70%
35	Hong,D.2011	South Korea	2000–2005	18	-	-	80.00%
36	Jayalekshmi, P.,2011	India	1991–1997	35	62.90%	36.40%	26.00%
37	Jiang X.2017	China	2002–2017	25	-	-	38.90%
38	JiIanjun LU,2019	China	2011–2013	74	-	-	84.85%
39	Jie Yin,2019	China	2000–2016	40	-	-	87.50%
40	Jin, F.1998	China	1988–1991	941	65.90%	47.20%	41.50%
41	K.Kritpracha.2008	Thailand	1987–1998	105	-	-	50.21%
42	Kaili.2012	China	2005–2007	335	-	-	65.50%
43	Kang S.2013	China	2002–2008	213	59.40%	57.80%	53.00%
44	Karabulut B.2005	Turkey	1999–2003	26	51.00%	-	-
45	Karimi Zarchi M,0.2015	Iran	2006–2012	120	-	-	83.34%
46	Khunnarong, J.2008	Thailand	1996–2003	170	-	-	54.90%
47	Ku-F-C.2017	Taiwan	2000–2013	891	-	-	37.50%
48	Kwang-Beom Lee.2006	South Korea	1997–2003	52	-	-	60.00%
49	Law, S. C.2011	China	1996–2001	1831	83.00%	69.00%	63.50%
50	Loka A.2002	Japan	1985–1994	1494	-	-	40.90%
51	Martin, N,2011	Thailand	1990–2000	193	68.40%	52.30%	48.50%
52	Matsuda T.2010	Japan	1993–1999	491	-	-	52.00%
53	Matsumoto H.2013	Japan	1998–2011	16	-	-	73.10%
54	Menczer J.2012	Israel	1994–1999	225	-	-	-
55	Min K.W.2012	South Korea	1995–2006	129	-	-	79.10%
56	Mok J.E.2006	South Korea	1993–2004	10	60.00%	-	42.00%
57	Nagase S.2019	Japan	2015 − 2010	752	-	-	88.50%
58	Nakagawa-Senda,2017	Japan	2006–2008	865	-	-	51.00%
59	Nakashima N.1989	Japan	1965–1987	71	-	-	69.30%
60	Natee J.2006	Thailand	1995–2004	43	-	-	85.20%
61	Pandey D.2004	India	1981–2000	58	-	73.10%	-
62	Park J.Y.2006	South Korea	2001–2005	46	-	66.63%	-
63	Piura B.1999	Israel	1978–1998	11	-	50.00%	-
64	R. Kobayashi.2017	Japan	1991–2014	110	-	-	88.20%
65	S.Kuntito.2012	Japan	1996–2004	31	-	69.20%	84.70%
66	Saito T.1995	Japan	2013–2013	138	-	-	74.20%
67	Sakai K.2011	Japan	1986–2009	180	-	-	62.90%
68	Sankaranarayananl R.1995	India	1982–1982	452	-	-	61.50%
69	Satoru Nagase,2019	Japan	2010–2015	7527	-	-	88.50%
70	Hasani S,2019	Iran	2011–2017	179	-	-	48.10%
71	Inoue S,2019	Japan	2006–2008	1309	50.50%	-	23.00%
72	Sozen H.2015	Turkey	1998–2010	50	-	-	92.00%
73	Sriplung, H.2011	Thailand	1990–1999	173	81.10%	56.20%	48.60%
74	Suh D.H.2015	South Korea	1995–2013	193	68.50%	81.10%	-
75	Suita S.2002	Japan	1975–2000	60	-	-	75.00%
76	Sumitsawan, Y.2011	Thailand	1993–1997	162	90.30%	71.10%	66.20%
77	Sun H.D.2011	Taiwan	1948–2010	167	-	-	96.50%
78	Surprasert P.2006	Thailand	1995–2005	1076	-	-	55.40%
79	Swaminathan, R., 2011	India	1990–1999	808	-	-	27.40%
80	Taek sang Lee.2013	South Korea	1997–2008	1032	-	-	94.50%
81	Taskin S.2013	Turkey	2001–2010	297	-	-	51.60%
82	Teramukai S.2007	Japan	1994–2000	768	-	-	54.00%
83	Terzi A.2013	Japan	1984–2001	156	-	90.60%	54.00%
84	Tong X.2008	China	1948–2007	76	-	-	91.80%
85	Tsubamoto H.2013	Japan	1996–2009	73	-	-	74.00%
86	Tsukuma, H., 2006	Japan	1993–1996	373	78.20%	56.20%	48.20%
87	Uegaki K.2014	Japan	2001–2011	51	-	-	85.70%
88	Uygun K.2003	Turkey	1979–1998	952	89.00%	84.00%	81.00%
89	Vandana Jain,2019	India	2004–2016	14	-	40.52%	33.00%
90	Vatanasapt, V. 1998	Thailand	1985–1992	253	57.50%	39.40%	34.00%
91	Veras E.2009	Japan	1985–2006	122	-	90.00%	68.00%
92	Wang P.H.2014	Taiwan	1994–2010	44	-	-	55.80%
93	Yamagami W,2019	Japan	2004–2008	9747	-	-	94.90%
94	Wong K.H.2012	Hong Kong	1997–2006	2941	-	-	63.10%
95	Xiang, Y. B. 2011	China	1992–1995	1087	66.40%	48.40%	42.70%
96	Xishan, H.2011	China	1991–1999	1124	77.20%	62.70%	59.70%
97	Y.M.Kim.2006	South Korea	1991–2004	35	-	-	92.00%
98	Yamagami W.2015	Japan	2007–2007	3681	-	-	31.90%
99	Yamagami W.2017	Japan	1975–2012	9384	-	-	90.50%
100	Yamamoto S.2011	Japan	1992–2003	254	83.40%	81.40%	68.80%
101	Yeole, B. B. 2011	India	1992–1994	2029	49.70%	29.10%	22.80%
102	Yong Kuei lim.2011	Singapore	2000–2009	75	-	-	84.00%
103	Yuk J.S.2018	Korea	2006–2010	78,826	-	95.70%	88.90%
104	Zeng H.2018	China	2003–2005	678	-	-	38.90%
105	Zhao Q.2017	China	2010–2015	50	-	-	83.60%
106	Zhao T l.2016	China	1997–2014	102	98.70%	96.40%	-
107	Zhao T l.2017	China	1997–2015	53	-	-	69.00%
108	Ziying Lei,2020	China	2010–2017	584	-	60.30%	-

### Quality Appraisal

The results of the quality assessment of manuscripts are shown in Appendix 2. Based on the review using the relevant checklist, 79 studies were determined to be of good quality, and 29 articles had average quality.

### Heterogeneity

The result of the chi-squared test and I^2^ index elucidated that there is significant heterogeneity between studies. According to the analysis, 1 year survival of ovarian cancer was: I^2^ = 99.93%, P < 0.001; 3 years was: I^2^ = 99.96%, P < 0.001); and 5 years was: I^2^ = 99.92%, P < 0.001). As a result, a random-effects model was used for all analysis.

### Results of the Meta-Analysis

First, the articles were sorted based on the year of publication, and then the survival was differentiated into 1, 3, and 5 years. Meta-regression was also completed based on the year of the study.

### 1-year Survival Rate of Ovarian Cancer in Asian Countries

**Fig. 2:**
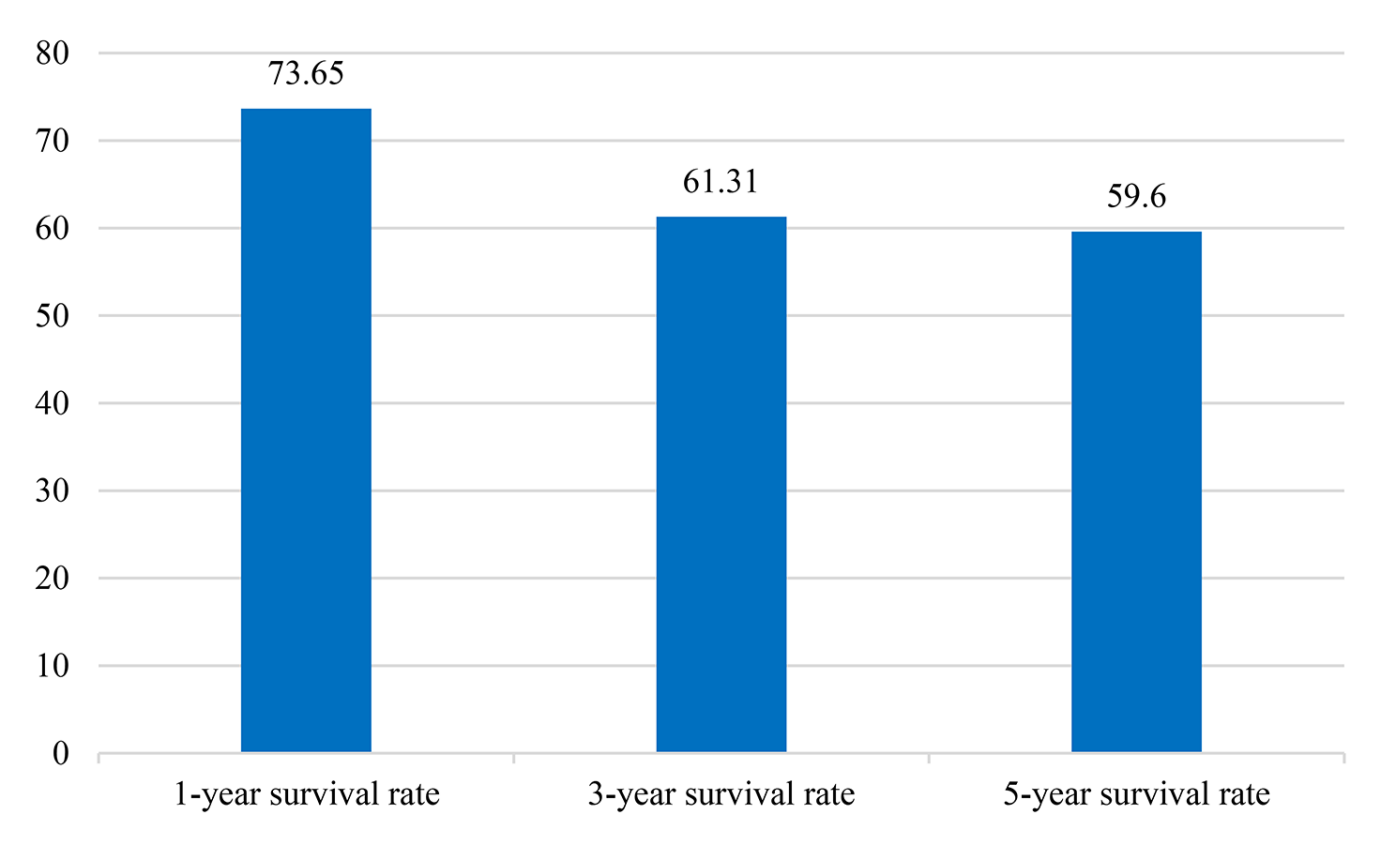
1,3 and 5-year Survival Rate of Ovarian Cancer in Asian Countries

From the total number of articles that were included in the final analysis of this paper, 41 studies showed that based on a random-effect model, the 1-year survival was 73.65% (95% CI, 68.66–78.64). (Fig. 2)

### 3-year Survival Rate of Ovarian Cancer in Asian Countries

From the total number of articles that were included in the final analysis of this paper, 50 studies showed that based on a random-effect model the 3-year survival was 61.31% (95% CI, 55.39–67.23).(Fig. 2).

### 5-year Survival Rate of Ovarian Cancer in Asian Countries

From the total number of articles that were included in the final analysis of this paper,159 showed that based on a random-effect model, the 5-year survival was 59.60% (95% CI, 56.06–63.13).(Fig. 2).

### Ovarian Cancer survival rate by Asian Country

**Fig. 3:**
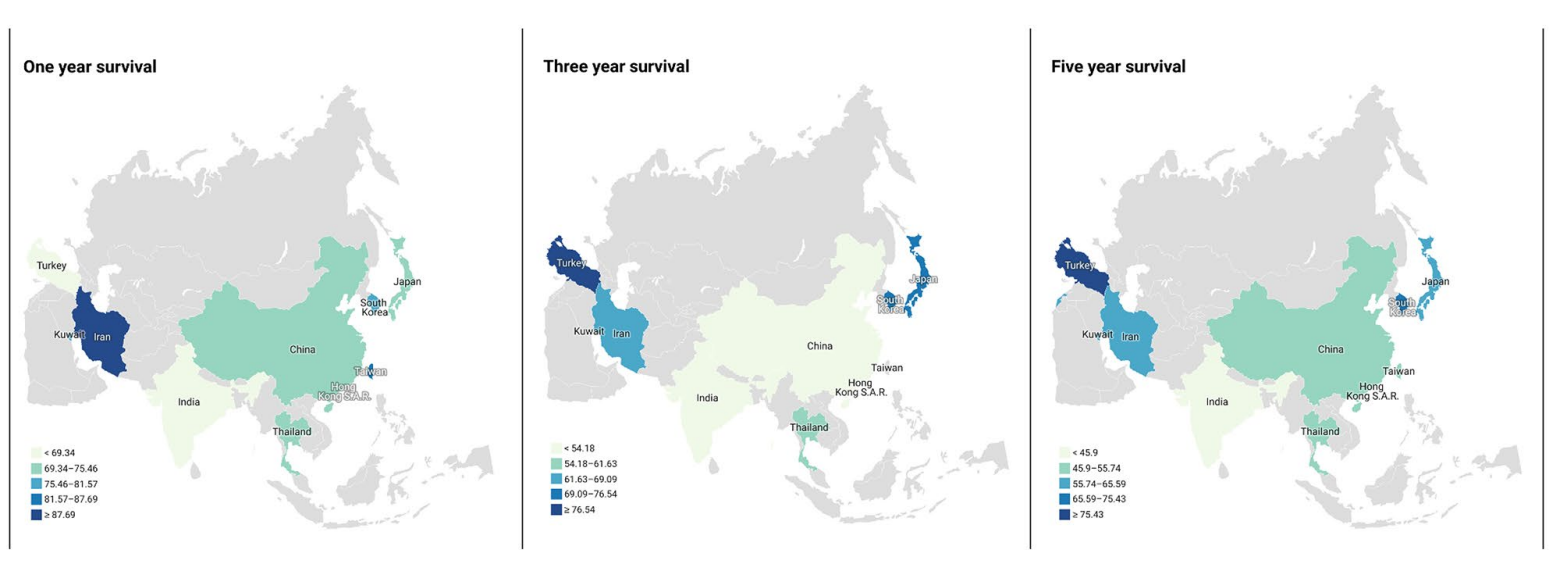
1-year survival rate, 3-year survival rate, and 5-year survival rate of ovarian Cancer in Asian Countries

Results of ovarian cancer survival rate in 12 countries are shown in Table 2; Fig. 3. The highest 1, 3, and 5 years survival rates are respectively in Iran (93.80), Turkey (84.0), and Turkey (85.27), and the lowest survival rates are respectively seen in Singapore (63.23) and India (46.72).

**Table 2:** Result of meta-analysis and heterogeneity of survival rate of ovarian Cancer in Asian Countries base on each country and year of survival

Country	Total	Year of Survival											
		1				3				5			
		N	Effect estimate	I^2^	P	N	Effect estimate	I^2^	P	N	Effect estimate	I^2^	P
China	56	9	72.56 (63.75, 81.37)	99.33	≤ 0.001	13	53.02 (43.65, 62.39)	99.29	≤ 0.001	34	54.75 (46.89, 62.61)	99.25	≤ 0.001
HongKong	13	3	82.17 (54.16, 110.19)	99.97	≤ 0.001	4	65.00 (40.95, 89.06)	99.91	≤ 0.001	6	59.83 (38.05, 81.62)	99.92	≤ 0.001
India	21	5	64.22 (48.32, 80.11)	99.50	≤ 0.001	7	46.72 ( 32.30, 61.14)	99.15	≤ 0.001	9	36.06 (23.59, 48.54)	99.22	≤ 0.001
Iran	13	1	93.80 (89.33, 98.27)	NR	NR	2	63.63 (21.30, 105.97)	98.67	≤ 0.001	10	60.39 (50.82, 69.96)	94.52	≤ 0.001
Israel	2	NR	NR	NR	NR	NR	NR	NR	NR	2	64.54 (50.64, 78.44)	17.57	0.271
Japan	57	4	71.89 (57.56, 86.22)	99.53	≤ 0.001	8	75.71 (58.90, 92.53)	99.64	≤ 0.001	45	63.87 (57.26, 70.48)	99.94	≤ 0.001
Korea	29	7	81.52 (70.97, 92.07)	99.65	≤ 0.001	8	72.69 (58.13, 87.26)	99.88	≤ 0.001	14	70.90 (58.88, 82.93)	99.83	≤ 0.001
Kuwait	2	1	79.00 (73.44, 84.55)	NR	NR	NR	NR	NR	NR	1	42.60 (35.89, 49.30)	NR	NR
Singapore	16	3	63.23 (41.84, 84.61)	99.99	≤ 0.001	3	55.74 (42.49, 68.99)	99.96	≤ 0.001	10	56.03 (45.71, 66.34)	99.92	≤ 0.001
Taiwan	11	1	87.00 (86.39, 87.60)	NR	NR	NR	NR	NR	NR	10	54.94 (37.98, 71.90)	71.94	≤ 0.001
Thailand	16	4	74.41 ( 60.27, 88.55)	95.57	≤ 0.001	4	54.66 ( 41.78, 67.54)	92.50	≤ 0.001	8	55.03 (44.95, 65.10)	99.75	≤ 0.001
Turkey	14	3	63.47 (36.03, 90.92)	93.79	≤ 0.001	1	84.00 (81.62, 86.37)	NR	≤ 0.001	10	85.27 (76.26, 94.28)	94.59	≤ 0.001
**Overall**	250	41	73.65 (68.66, 78.64)	99.93	≤ 0.001	50	61.31 (55.39, 67.23)	99.96	≤ 0.001	159	59.60 (56.06, 63.13)	98.88	≤ 0.001

*NR; Not reported

### Metaregression Ovarian Cancer Survival Rate in Asian Countries

**Fig. 4:**
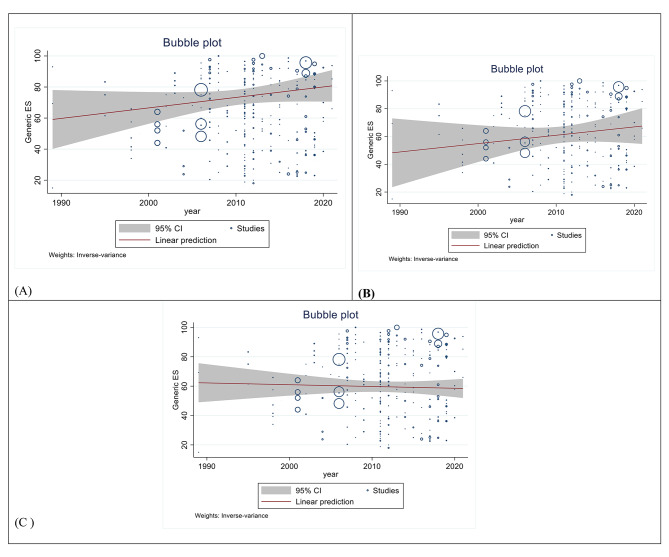
Bubble plot of standard error by point estimate for assessment of meta-regression (1, 3, and 5-year Ovarian cancer survival rate) [**A**: 1-year survival rate, **B**: 3-year survival rate, **C**: 5-year survival rate]

Although in the recent years, the 1-year survival (Reg Coef = 0.6756, p = 0.119) and 3-year survival (Reg Coef = 0.6012, p = 0.287) has increased, this increase was not statistically significant. Also the 5 year survival rate has decreased (Reg Coef = − 0.1205, p = 0.678), while this decerease was also not signifant. (Fig. 4)

### Publication Bias

Ultimately, we chose the funnel plots to evaluate the release bias for 1, 3, and 5 years ovarian cancer survival rate in Asian countries. The results of the egger test confirmed this bias (Appendix 3).

Bias for 1 year: 1.86, 95% CI = -11.14 to 14.87; P = 0.7738.

Bias for 3 years: 2.40, 95% CI = -11.76 to 16.56; P = 0.7346.

Bias for 5 years: 2.64, 95% CI = -2.16 to 7.46; P = 0.2795.

## Discussion

In the present study, we conducted a meta-analysis to evaluate the 1, 3 and 5 year survival rate of ovarian cancer across 12 Asian countries. The mean 1-year survival in this study was estimated to be 73.65%. According to the results, Iran’s 1-year survival rate was estimated to be the highest and was 93.80% and Singapore’s 1-year survival rate was evaluated to be the lowest and was 63.23%. In a 2013 cohort study of women with ovarian cancer in Denmark, Grann et al. found that the overall 1-year survival rate between 2000 and 2002 was 73% and between 2009 and 2011 was 69% [14]. In a 2012 study, the 1-year survival rate of ovarian cancer between 1978 and 2002 was evaluated to be 74.6% in Finland, 75.6% in Norway, 79.3% in Sweden, 60.7% in Ireland., 60.7% in England, 62.9% in Northern Ireland, 60.8% in Scotland, 62.9% in Wales, 70.9% in Austria, 69.7% in Germany, 69.5% in the Netherlands, 78.5% in Switzerland, 64.9% in the Czech Republic, 4.64% in Poland, 1.56% in Slovakia, 69.6% in Italy, 68.8% in Slovenia and 63.6% in Spain [15]. In 2017, another study by Stewart et al. reviewed ovarian cancer survival rate between 2001 and 2009 in 37 states, which includes 80% of the population of the United States. The survival rate for ovarian cancer in women between 15 and 90 years old was 72.3% from 2001 to 2003 and increased to 73.3% from 2004 to 2009 [16]. The estimated 1-year survival rate in the present study is higher than in the previous studies. Only Finland, Sweden, and Switzerland have slightly higher survival rates.

The 3-year survival rate of ovarian cancer in this study was estimated to be 61.31%. The highest and lowest 3-year survival rates in our study were found to be 84% in Turkey and 46.72% in India respectively. According to a study published in 2014 by Anuradha et al., women with invasive epithelial ovarian cancer in 2005 had a 3-year cancer survival rate of 57% in Western Australia and 50% in South Australia [17]. Cabasag et al. studied 3-year survival rate of ovarian cancer between 2014 and 2010 in Australia, Canada, Denmark, Ireland, New Zealand, Norway, and the United Kingdom where the results were 56.4%, 50.1%, 53.6%, 44.8%, 45.5%, 57.2%, and 47.3% respectively [18].

The 5-year survival rate of ovarian cancer in the present study is 59.60%. Turkey, with 85.27%, has the highest, and India, with 36.06%, has the lowest 5-year survival rate. In the United States, the incidence of ovarian cancer is relatively low, with a 5-year survival rate of 53% in patients undergoing surgery and 8% in patients that do not undergo surgery. In Canada, the 5-year survival rate of ovarian cancer was estimated to be between 37 and 43%, and in Japan, the United Kingdom, France, and Sweden was respectively 55%, 43%, 43%, and 45% [19]. Studies which were conducted in the Netherlands and Korea illustrated that the 5-year survival rate in the Netherlands rose from 18% to 1993 to 28% in 2004, and reported the 5-year survival rate of ovarian cancer in Korea in 2011 to be 60% [20, 21].

From 2000 to 2007, the 5-year survival rate for European women with ovarian cancer was 38%. However, the 5-year survival rate for this cancer was lower in Ireland and the United Kingdom, where it was reported to be 31%, as compared to the results of our current study. Moreover, in Eastern Europe, Southern Europe, Central Europe, and Northern Europe, the 5-year survival rate were estimated to be 34.4%, 38%, 40.5%, and 41.1% respectively [22]. In Western and Southern Australia, the 5-year survival of invasive epithelial ovarian cancer is estimated to be 46% and 40%, respectively. Additionally, the 5-year survival rate of ovarian cancer between 2003 and 2008 was approximately 44% in United States, 43% in England, 45% in Canada, 55% in Japan, 37% in Denmark, and 45% Sweden [17]. Srivastava et al. found that the 5-year survival rate in Caucasian women has surged from 40.7 to 45% from 1992 to 2008; however, the 5-year survival rate decreased in African American women from 47.9 to 40.3% over the same period and decreased to 36% from 2006 to 2012 [23]. In a 2020 study, Bian et al. studied the effects of a previous malignancy on ovarian cancer survival rate between 2004 and 2015. They reported a 5-year survival rate of ovarian cancer with a previous malignancy to be 35.1%, and with no previous malignancy to be 43.2% [24]. A retrospective study conducted in China, which analyzed 63 pathological cases of ovarian cancer from 2000 to 2018, reported a 5-year survival rate of 69% in patients who underwent surgery for treatment. The study also found an overall 5-year survival rate of 80% for all patients [25].

A 2020 cohort study by Beachler et al. reported an overall 5-year survival rate of 47.7% in advanced ovarian cancer patients in the United States from 2018 to 2010 [26].

The findings of our study indicate that the survival rate of ovarian cancer is higher in Asian countries when compared to those in Europe, America, Australia, and Africa.

Ovarian cancer is generally less common in Asia and the Middle East and has better outcomes than in the United States and Europe. Ovarian cancer is also diagnosed in women in Asia and the Middle East at a younger age, which may be a contributing factor to better survival rates [19]. Generally, this difference in survival could be related to different risk factors, increased cancer incidence, or more specific reporting of the death rates, which needs to be thoroughly investigated in future studies. It can also be caused by different treatment methods such as the use of lymphadenectomy [27, 28], which can lead to different survival in patients in different regions.

### The strength and limitations

The type and quality of the studies included in this study are among the study’s limitations. Also, the volume of sample studies and the number of studies conducted in each country can affect the results of the present study. In addition, more than half of Asian countries have not published any studies on ovarian cancer survival rate, so more accurate studies are needed for precise assessment, especially in unreported countries. The power of the present study is the introduction of observational studies with follow-up design (cohort) and meta-regression analysis to identify heterogeneity sources.

## Conclusion

Ovarian cancer can be one of the most important cancers among women and can be fatal if diagnosed late. The survival rate of ovarian cancer in the present study shows that in most cases, Asian countries have a higher survival rate than European countries, and these results can be a basis for developing treatment strategies and health interventions. One of the reasons for the higher survival of this cancer in Asian countries can be due to the difference in the type of cancer as well as the cancer BIRAD. Also, the difference in diet and lifestyle in Asian countries compared to European countries can be another reason for the difference in survival in these two continents. It seems that a healthier life than an industrial life can have an effect on cancer survival. However, it is highly preventable by recognizing risk factors. With improved disease management, early detection and better treatment, ovarian cancer mortality can be better managed and even prevented. It is also recommended for future studies to investigate different treatments and make better decisions for more effective treatment that can bring more survival for patients. In addition, it is recommended to investigate risk factors, genetic differences, differences in the type of cancer and degree of cancer, and nutritional differences for further studies.

## Electronic supplementary material

Below is the link to the electronic supplementary material.

Supplementary Material 1

Supplementary Material 2

Supplementary Material 3

## Data Availability

The data that support the findings of this study are available from the corresponding author, [HGH], upon reasonable request.
