# Supplementary material for: Survival rate of ovarian cancer in Asian countries: a systematic review and meta-analysis
Source: BMC Cancer. 2023 Jun 16;23:558. doi: 10.1186/s12885-023-11041-8 (PMC10276364; doi:10.1186/s12885-023-11041-8)
Supplement: Supplementary file 2 — Supplementary Material 2 [file 12885_2023_11041_MOESM2_ESM.docx]

| **Appendix 2:** Newcastle-Ottawa Quality Assessment Form for Cohort Studies | | | | | |
| --- | --- | --- | --- | --- | --- |
| Quality^*^ | Total | Outcome | Comparability | Selection | Author (year) |
| Good | 7 | 3 | 2 | 2 | Khalafi-Nezhad A,2021 |
| Good | 7 | 3 | 1 | 3 | Aoki D.2014 |
| Fair | 6 | 3 | 1 | 2 | Arab, M..2009 |
| Good | 7 | 3 | 1 | 3 | Ayhan A.2008 |
| Good | 7 | 3 | 1 | 3 | Bhika B.2004 |
| Fair | 6 | 3 | 1 | 2 | Bi R.2016 |
| Fair | 6 | 3 | 1 | 2 | Bian,C.2015 |
| Fair | 6 | 3 | 1 | 2 | Bozkaya, Y.2017 |
| Good | 7 | 3 | 1 | 3 | C.Li.2012 |
| Good | 7 | 3 | 1 | 3 | Chay W.Y.2013 |
| Fair | 6 | 3 | 1 | 2 | Chen C.A.2018 |
| Good | 7 | 3 | 1 | 3 | Chen S.2014 |
| Fair | 6 | 3 | 1 | 2 | Chen, J. G. 2011 |
| Good | 6 | 2 | 1 | 3 | Chen,M.C.2018 |
| Good | 7 | 3 | 1 | 3 | Chen,Y .2015 |
| Good | 7 | 3 | 1 | 3 | Chia, K. S. , 2011 |
| Fair | 6 | 3 | 1 | 2 | Chia, K. S., 2001 |
| Good | 6 | 2 | 1 | 3 | Chul Chun,K.2011 |
| Good | 7 | 3 | 1 | 3 | Chung,H.H.2007 |
| Good | 7 | 3 | 1 | 3 | Dan Nie.2019 |
| Good | 7 | 3 | 1 | 3 | Dikshit, R. 2011 |
| Fair | 6 | 3 | 1 | 2 | E. Alawadhi,2019 |
| Good | 6 | 2 | 1 | 3 | Egemen Ertas,I.2014 |
| Fair | 6 | 3 | 1 | 2 | Gaemmaghami, F.2011 |
| Good | 6 | 2 | 1 | 3 | Gaurav Das,2020 |
| Fair | 6 | 3 | 1 | 2 | Gek-Hsiang Lim.2009 |
| Good | 6 | 2 | 1 | 3 | Ghaemmaghami, F.2008 |
| Good | 7 | 3 | 1 | 3 | Guangquan Liu.2017 |
| Good | 7 | 3 | 1 | 3 | Gue,J.2018 |
| Fair | 6 | 3 | 1 | 2 | Guvenal, T.2013 |
| Good | 7 | 3 | 1 | 3 | Han,Y.2016 |
| Good | 6 | 2 | 1 | 3 | Hee-Beom Yang,2020 |
| Fair | 6 | 3 | 1 | 2 | Helpman, L.2005 |
| Good | 6 | 2 | 1 | 3 | Higash, M.2011 |
| Good | 7 | 3 | 1 | 3 | Hong,D.2011 |
| Good | 7 | 3 | 1 | 3 | Jayalekshmi, P.,2011 |
| Fair | 6 | 3 | 1 | 2 | Jiang X.2017 |
| Good | 7 | 3 | 1 | 3 | JiIanjun LU,2019 |
| Fair | 6 | 3 | 1 | 2 | Jie Yin,2019 |
| Fair | 6 | 3 | 1 | 2 | Jin, F.1998 |
| Good | 6 | 2 | 1 | 3 | K.Kritpracha.2008 |
| Good | 6 | 2 | 1 | 3 | Kaili.2012 |
| Good | 7 | 3 | 1 | 3 | Kang S.2013 |
| Good | 6 | 2 | 1 | 3 | Karabulut B.2005 |
| Good | 7 | 3 | 1 | 3 | Karimi Zarchi M,.2015 |
| Good | 5 | 1 | 1 | 3 | Khunnarong, J.2008 |
| Good | 7 | 3 | 1 | 3 | Ku-F-C.2017 |
| Good | 7 | 3 | 1 | 3 | Kwang-Beom Lee.2006 |
| Good | 7 | 3 | 1 | 3 | Law, S. C.2011 |
| Fair | 6 | 3 | 1 | 2 | Loka A.2002 |
| Good | 7 | 3 | 1 | 3 | Martin, N,2011 |
| Fair | 6 | 3 | 1 | 2 | Matsuda T.2010 |
| Fair | 6 | 3 | 1 | 2 | Matsumoto H.2013 |
| Good | 6 | 2 | 1 | 3 | Menczer J.2012 |
| Fair | 5 | 2 | 1 | 2 | Min K.W.2012 |
| Fair | 6 | 3 | 1 | 2 | Mok J.E.2006 |
| Fair | 5 | 2 | 1 | 2 | Nagase S.2019 |
| Fair | 6 | 3 | 1 | 2 | Nakagawa-Senda,2017 |
| Fair | 6 | 3 | 1 | 2 | Nakashima N.1989 |
| Good | 6 | 2 | 1 | 3 | Natee J.2006 |
| Fair | 6 | 3 | 1 | 2 | Pandey D.2004 |
| Good | 7 | 3 | 1 | 3 | Park J.Y.2006 |
| Good | 7 | 3 | 1 | 3 | Piura B.1999 |
| Good | 7 | 3 | 1 | 3 | R. Kobayashi.2017 |
| Good | 7 | 3 | 1 | 3 | S.Kuntito.2012 |
| Good | 7 | 3 | 1 | 3 | Saito T.1995 |
| Fair | 6 | 3 | 1 | 2 | Sakai K.2011 |
| Good | 6 | 2 | 1 | 3 | Sankaranarayananl R.1995 |
| Good | 6 | 2 | 1 | 3 | Satoru Nagase,2019 |
| Good | 6 | 2 | 1 | 3 | Hasani S,2019 |
| Good | 6 | 2 | 1 | 3 | Inoue S,2019 |
| Good | 6 | 2 | 1 | 3 | Sozen H.2015 |
| Good | 7 | 3 | 1 | 3 | Sriplung, H.2011 |
| Fair | 6 | 3 | 1 | 2 | Suh D.H.2015 |
| Good | 7 | 3 | 1 | 3 | Suita S.2002 |
| Fair | 6 | 3 | 1 | 2 | Sumitsawan, Y.2011 |
| Good | 7 | 3 | 1 | 3 | Sun H.D.2011 |
| Good | 7 | 3 | 1 | 3 | Surprasert P.2006 |
| Good | 7 | 3 | 1 | 3 | Swaminathan, R., 2011 |
| Good | 7 | 3 | 1 | 3 | Taek sang Lee.2013 |
| Good | 7 | 3 | 1 | 3 | Taskin S.2013 |
| Good | 7 | 3 | 1 | 3 | Teramukai S.2007 |
| Good | 7 | 3 | 1 | 3 | Terzi A.2013 |
| Good | 7 | 3 | 1 | 3 | Tong X.2008 |
| Fair | 6 | 3 | 1 | 2 | Tsubamoto H.2013 |
| Good | 7 | 3 | 1 | 3 | Tsukuma, H., 2006 |
| Good | 7 | 3 | 1 | 3 | Uegaki K.2014 |
| Good | 7 | 3 | 1 | 3 | Uygun K.2003 |
| Good | 7 | 3 | 1 | 3 | Vandana Jain,2019 |
| Good | 7 | 3 | 1 | 3 | Vatanasapt, V. 1998 |
| Good | 7 | 3 | 1 | 3 | Veras E.2009 |
| Good | 7 | 3 | 1 | 3 | Wang P.H.2014 |
| Fair | 6 | 3 | 1 | 2 | Yamagami W,2019 |
| Good | 7 | 3 | 1 | 3 | Wong K.H.2012 |
| Good | 7 | 3 | 1 | 3 | Xiang, Y. B. 2011 |
| Good | 7 | 3 | 1 | 3 | Xishan, H.2011 |
| Good | 7 | 3 | 1 | 3 | Y.M.Kim.2006 |
| Good | 7 | 3 | 1 | 3 | Yamagami W.2015 |
| Good | 7 | 3 | 1 | 3 | Yamagami W.2017 |
| Good | 7 | 3 | 1 | 3 | Yamamoto S.2011 |
| Good | 7 | 3 | 1 | 3 | Yeole, B. B. 2011 |
| Good | 7 | 3 | 1 | 3 | Yong Kuei lim.2011 |
| Good | 7 | 3 | 1 | 3 | Yuk J.S.2018 |
| Good | 7 | 3 | 1 | 3 | Zeng H.2018 |
| Good | 7 | 3 | 1 | 3 | Zhao Q.2017 |
| Good | 7 | 3 | 1 | 3 | Zhao T l.2016 |
| Good | 7 | 3 | 1 | 3 | Zhao T l.2017 |
| Good | 7 | 3 | 1 | 3 | Ziying Lei,2020 |

* **Newcastle-Ottawa Quality Assessment Form for Cohort Studies**
