# Supplementary material for: Survival rate of ovarian cancer in Asian countries: a systematic review and meta-analysis
Source: BMC Cancer. 2023 Jun 16;23:558. doi: 10.1186/s12885-023-11041-8 (PMC10276364; doi:10.1186/s12885-023-11041-8)
Supplement: Supplementary file 3 — Supplementary Material 3 [file 12885_2023_11041_MOESM3_ESM.docx]

A:Publication bias 1 years

B: Publication bias 3 years

C: Publication bias 5 years

**Appendix 3:** Funnel plot of standard error by point estimate for assessment of publication bias
